# Differential Expression and Prognostic Value of Cytoplasmic and Nuclear Cyclin D1 in Prostate Cancer

**DOI:** 10.1155/2020/1692658

**Published:** 2020-05-30

**Authors:** Zhi Cao, Xi Chen, Yalong Xu, Fei Guo, Jin Ji, Huan Xu, Jingyi He, Yinghao Sun, Fubo Wang

**Affiliations:** Department of Urology, Changhai Hospital, Navy Medical University, Shanghai, China

## Abstract

Cyclin D1 (CCND1) has been revealed as a key regulating protein in cell cycle (G1 phase) and plays a critical role in promoting tumor development. The purpose of our study was to investigate the associations between CCND1 and biochemical recurrence of prostate cancer (PCa). We performed immunostaining of CCND1 on a tissue microarray and evaluated the CCND1 expression levels based on the intensity and extent of staining. The clinical data was collected, and the follow-up data was received by searching our follow-up database called “PC-follow”. We revealed that CCND1 expression patterns were different between cytoplasm and nucleus in this study, and the expression of CCND1 in adjacent normal tissues was higher than that in PCa tissues (*P* < 0.001), while nuclear CCND1 showed the opposite distribution characteristic (*P* < 0.001). The cytoplasmic CCND1 also showed correlation with several clinical factors, e.g., tumor T stage (*P* < 0.001), Gleason score (*P* = 0.028), positive surgical margin (*P* = 0.037), and capsule invasion (*P* = 0.04). We also revealed that cytoplasmic CCND1 is a protective prognostic factor in the biochemical recurrence (BCR) free time analysis (*P* = 0.002). However, the nuclear CCND1 showed no correlation with clinical factors or prognostic value in this study. This study found that cytoplasmic and nuclear CCND1 have significant different expression patterns in PCa tissues, and cytoplasmic CCND1 has a certain prognostic value in the BCR analysis.

## 1. Introduction

Prostate cancer (PCa) is a serious health threat for males in the United States and Europe with the highest morbidity and the second fatality rate among all kinds of tumors according to the latest report [1]. Despite PCa is the sixth-highest morbidity and seventh-highest mortality cancer in China [2], this threat is raising rapidly, the incidence rate of PCa increased from 1.6 × 10 − 5 to 4.3 × 10 − 5 from 2002 to 2008 [3] which makes it a serious health concern in China. Radical prostatectomy (RP) is one of the most effective treatments for localized PCa; however, the risk of early biochemical recurrence (BCR) occurred in patients performed RP is almost 20% [4]. Thus, there is great significance for doctors to identify these higher-risk patients as early as possible and take further adjuvant therapy like androgen deprivation therapy or external beam radiotherapy to prolong their survival time. Several clinical indexes and molecular biomarkers have recently been reported to predict the BCR after RP and guide further clinical treatment [5, 6], yet there is plenty of room for research in this area.

Cyclin D1 (CCND1) is a key regulating factor in cell cycle (G1 phase) encoded by chromosome 11q13 CCND1 gene, firstly reported in 1991 [7]. It has been reported to be a regulating subunit of cyclin-dependent kinase (Cdk) [8]. Specifically, the CCND1 Cdk4 complex phosphorylates the transcriptional repressors which trigger the E2F-dependent transcription, which is crucial in S phase entry [8]. Besides, This molecule could also regulate the process by a Cdk independent pathway [9]. In both ways, overexpression of CCND1 results in a shorter cell cycle and tumor progression. The overexpression of cytoplasmic CCND1 is also reported to be associated with the tumor invasive capability [10]. Thus, CCND1 plays a critical role in promoting tumor development.

It is worth noting that in various studies, different subcellular distributions of CCND1 have been revealed by immunohistochemistry [11]. The prognostic value of CCND1 in different subcellular distributions has been revealed in several different tumors as well [12–14]. There are several studies focusing on the prognosis value of nuclear CCND1 in prostate cancer [15–18], while the studies have noticed the cytoplasmic CCND1 [10, 14, 19] are very limited. And the value of cytoplasmic CCND1 expression in BCR prediction has never been evaluated according to our knowledge. To further determine the prognostic value of CCND1 for PCa patients underwent radical prostatectomy, we used several online sequencing databases and immunohistochemistry analysis (IHC) on tissue microarray (TMA) slides in this study.

## 2. Methods and Materials

### 2.1. Gene Sequencing Data Acquisition

Two gene-sequencing GEO databases (GSE21034 and GSE62872) were downloaded for analyzing the CCND1 gene expression level between PCa and non-PCa tissues. A previous PCa sequencing data of our institution including 272 samples was also enrolled and analyzed in this study to further explore the expression level of CCND1 between tumor tissue and adjacent normal tissue.

### 2.2. Tissue Microarray

This study was approved by the Ethics Committee of Changhai Hospital, Naval Medical University (Second Military Medical University). A total of 188 pairs of samples (tumors and adjacent tissues) of patients who had a radical prostatectomy in the Department of Urology, Changhai Hospital, from October 2002 to December 2008 were collected; the adjacent tissue was defined as the normal prostate tissue within 2 cm of the tumor. None of these patients received radiotherapy or hormonal therapy preoperatively. The original hematoxylin and eosin (H&E)-stained prostatectomy specimen slides were read by two pathologists, respectively, and the stages of prostate cancer were determined by the AJCC 2002 system. The clinical and pathological data of these patients were abstracted from their medical records and pathology reports.

The formalin-fixed postoperative specimens were obtained from the archive's office, and two cores (1 mm in diameter) from the noninflammatory area of each sample were transferred to the recipient block. The cores were then reconfirmed whether it can represent the diagnostic area after being cut into serial 3 mm sections.

### 2.3. Follow-Up Data Acquisition

The patients involved in this study were suggested to take serum PSA test every 3 months in the first year and every 6 months from the second year to the fifth year. Follow-up data were obtained by searching a database called “PC-follow” in the authors' department and querying the patients or relatives by phone calls. BCR was defined as serum PSA levels continuously exceeding 0.2 ng/ml, and the date of BCR was set as the date elevated serum PSA levels were firstly detected.

### 2.4. IHC and Scoring

We used CCND1 rabbit monoclonal antihuman antibody (dilution 1 : 100; Abcam, Cambridge, UK) to perform IHC on the 3 *μ*m TMA sections. The dewaxing and hydrating were performed on Leica AutoStainer XL (Wetzlar, Germany). The subsequent steps were performed using the maxim ready-to-use IHC kit (UltraSensitiveTM SP(Mouse/Rabbit), Maxim, Fuzhou, China) following the manufacturer's instructions, in which the heat antigen retrieval process was performed by using citrate buffer (1 mmol/l, pH 6.0). The stained slides were scanned by Pannoramic MIDI (3D HISTECH, Budapest, Hungary) and observed by case viewer software (3D HISTECH, Budapest, Hungary). Two independent pathologists who were not aware of the clinical data scored the slides basing on the intensity of staining and the range of staining, and the discrepant results were reevaluated until a consensus was made. A scoring system called “IE score” was used, in particular, this score was calculated as intensity score (0 − 3) × extent score (0 − 4) and ranged from 0 to 12. The IE score was further divided into two groups, representing weak (score 0-6) and strong (score 7-12) staining in this study.

### 2.5. Statistical Analysis

Statistical analysis was performed by SPSS 23.0 and SPSS 19.0 software (IBM, Armonk, USA). The comparison between tumors and adjacent normal tissues was performed by Wilcoxon signed-rank test and paired-sample *t* test. The gene expression level comparison between PCa and non-PCa tissues and the gene expression correlation between T stages in online data databases were performed by independent-sample *t* test. The gene expression correlation among Gleason scores was then measured by variance analysis. In the TMA data analysis, Mann-Whitney test was used in evaluating the correlation between T stage, N stage, prostate capsule invasion, surgical margin, and the IE score. The association between the postoperative Gleason score, PSA level on admission, and IE score were analyzed by Kruskal–Wallis *H* test. And the correlation between IE score and PSA level was revealed by Spearman's rank correlation. The evaluation of the impact of CCND1 IE score in the BCR free survival time was performed by the Kaplan–Meier method, and the significance was determined by the log-rank test. The free survival time of BCR in this study was defined as from the surgery date to the date of the first BCR observation or the date of the last follow-up. The prognostic value of the CCND1 expression and clinical values was evaluated by the univariate and multivariate Cox proportional hazard regression analysis, respectively.

## 3. Results

### 3.1. CCND1 mRNA Expression

**Figure 1:**
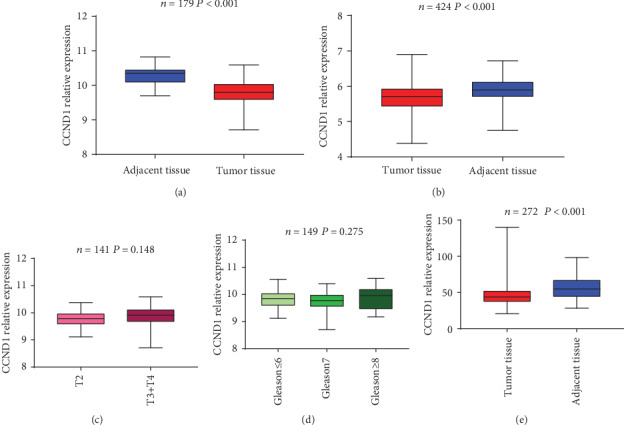
mRNA expression of CCND1 in online databases and our sequencing data. Comparison of CCND1 expression pattern between PCa and adjacent normal tissues in online databases GSE21034 (a) and GSE62872 (b). Different CCND1 mRNA expression patterns among tumor stages (c) and Gleason scores (d). Comparison of CCND1 expression pattern between PCa and adjacent normal tissues in our sequencing data (e).

In the GEO databases, CCND1 expression in adjacent normal tissues was significantly higher than the PCa tissues (GSE21034 *P* < 0.001Figure 1(a), GSE62872 *P* < 0.001Figure 1(b)). However, no statistical significance was found in the comparison of CCND1 expression with the T stage of tumor (GSE21034 *P* = 0.148Figure 1(c)) or Gleason score (GSE21034 *P* = 0.257Figure 1(d)). The previous PCa sequencing data of our institution explored that CCND1 expression level in adjacent normal tissues was higher than that in tumor tissues (*P* < 0.001Figure 1(e)).

### 3.2. Patient Description

A total of 188 patients in TMA aged 46-83 years (mean ± s.d., 66.8 ± 7.2 years) were included in this study. The clinical and pathological characteristics were described in Table 1. However, for the information missing in the medical record system, some clinical data of patients was incomplete.

### 3.3. CCND1 Protein Expression in TMA

**Figure 2:**
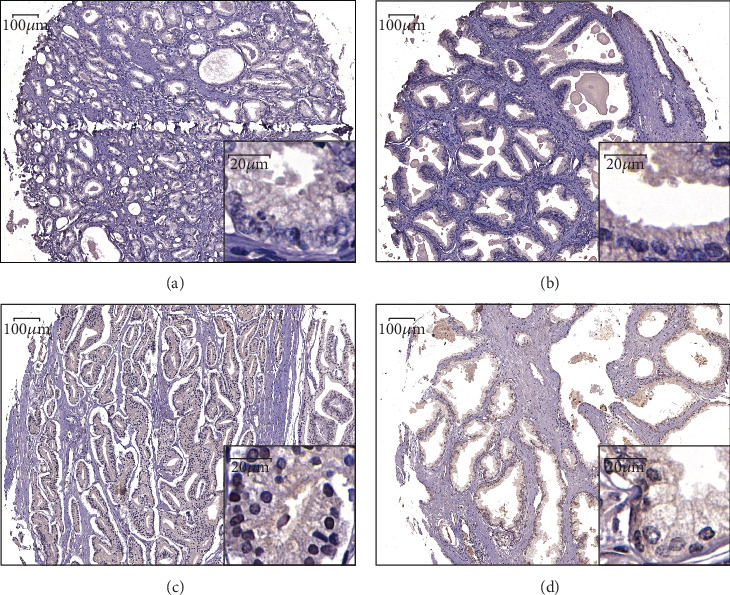
Examples of CCND1 immunostaining in PCa and adjacent normal tissues. Weak staining of cytoplasmic CCND1 in PCa tissue (a). Strong staining of cytoplasmic CCND1 in adjacent normal tissue (b). Strong staining of nuclear CCND1 in PCa tissue (c). Weak staining of cytoplasmic CCND1 in adjacent normal tissue (d).

**Figure 3:**
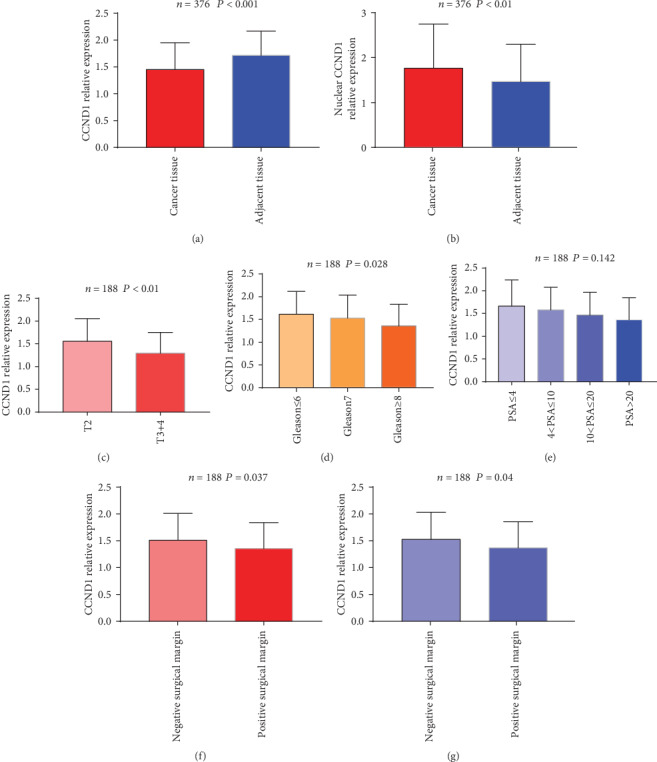
Expression of cytoplasmic and nuclear CCND1 in TMA. Comparison of cytoplasmic (a) and nuclear (b) CCND1 protein expression patterns between PCa and adjacent normal tissues in TMA. Cytoplasmic CCND1 protein expression patterns among tumor stages (c), Gleason scores (d), and different PSA levels (e). Cytoplasmic CCND1 protein expression patterns in tumors that had positive surgical margin (f) or capsule invasion (g).

In this study, both nuclear and cytoplasmic CCND1 staining was commonly found in the prostate epithelial cells, representative CCND1 staining patterns are shown in Figure 2. Similar to previous sequencing result, cytoplasmic CCND1 was found having a higher expression in adjacent normal tissue (*P* < 0.001Figure 3(a)). What is more, tumors with higher T stage (*P* < 0.001Figure 3(c)) and Gleason score (*P* = 0.028Figure 3(d)) had a significantly lower cytoplasmic CCND1 expression level. Tumors that had a positive surgical margin (*P* = 0.037Figure 3(f)) or capsule invasion (*P* = 0.04Figure 3(g)) also expressed a lower level of cytoplasmic CCND1. Although the PSA level did not show a statistical difference in different cytoplasmic CCND1 tumors (*P* = 0.142Figure 3(e)), cytoplasmic CCND1 had a negative correlation with PSA level on admission (Pearson correlation = −0.169, *P* = 0.01). However, no statistical significance was found between cytoplasmic CCND1 expression and pathological N stage (*P* = 0.177), seminal vesical invasion (*P* = 0.294), or nerve invasion (*P* = 0.285) (Table 2).

In contrast to cytoplasmic CCND1, nulcear CCND1 was found having a higher expression in tumor tissue (*P* < 0.001Figure 3(b)). Unfortunately, none of T stage (*P* = 0.931), N stage (*P* = 0.425), Gleason score (*P* = 0.4), PSA level (*P* = 0.63), positive surgical margin (*P* = 0.945), capsule invasion (*P* = 0.682), seminal vesical invasion (*P* = 0.811) or nerve invasion (*P* = 0.324) (Table 2) was found to have any correlation with nulcear CCND1 expression.

### 3.4. CCND1 Expression and BCR-Free Survival Time

**Figure 4:**
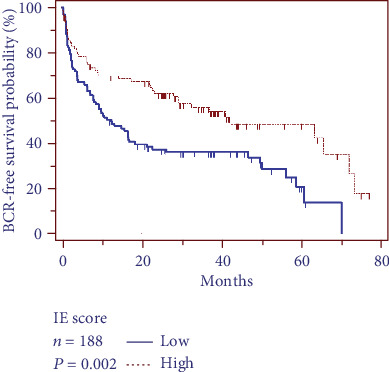
Relationship between cytoplastic CCND1 expression and patient prognosis. Log-rank test showed that patients with higher cytoplasmic CCND1 expression level have a longer BCR-free survival time.

Follow-up data were available in all 188 patients with a median follow-up time of 22.6 ± 5.74 months. There was a total of 61 patients (32.44%) experienced BCR in this study. Patients with higher cytoplasmic CCND1 expression level appeared to experience a longer BCR-free survival time in log-rank test (*P* = 0.002Figure 4). The univariate Cox proportional hazards regression showed that cytoplasmic CCND1 expression level was significantly associated with the time to BCR as a protective factor (*P* = 0.002). Nevertheless, in the multivariate Cox proportional hazard regression analysis, the Gleason score (*P* = 0.001), PSA level (*P* = 0.016), T stage (*P* = 0.012), and N stage (*P* < 0.001) showed as risk factors significantly associating with BCR time, while cytoplasmic CCND1 expression level was excluded in the equation (*P* = 0.242Table 3).

However, the nuclear CCND1 expression level was not associated with the time to BCR in the univariate Cox proportional hazards regression (*P* = 0.286Table 3). To further analyze the comprehensive influence of cytoplasm and nuclear CCND1 together to the BCR, we divided the patients into four groups (Figure 1S). However, the combination of cytoplasm and nuclear CCND1 did not show a better prognostic value than the cytoplasmic CCND1 alone.

## 4. Discussion

In this study, we revealed the expression pattern of CCND1 in prostate tumor and adjacent normal prostate tissues via using the IHC result of TMA and analyzing sequencing data. It is worth noting that we found the expression pattern of cytoplasmic and nuclear CCND1 were obviously different in the IHC result of TMA; therefore, we scored and analyzed the different expression pattern of CCND1, respectively.

Although CCND1 has been reported for almost 30 years, only a few studies have noticed the cytoplasmic CCND1 in PCa. Comstock et al. [14] demonstrated that positive cytoplasmic cyclin D1 was predominant in lower grade tumors and associated with lower PSA level in a study involved 179 samples. What is more, they also claimed that tumors with positive cytoplasmic cyclin D1 had the lowest Ki-67 index, which indicated that positive cytoplasmic cyclin D1 could be an indicator of good prognosis in PCa. Similarly, we found that tumor tissue had a lower expression level of cytoplasmic CCND1 than adjacent normal tissue, and tumors with higher cytoplasmic CCND1 had lower Gleason score (*P* = 0.028). Although the PSA level did not show a statistic difference, it can be seen from the bar chart that cytoplasmic CCND1 had a negative correlation with PSA level, which could be also be proved by Pearson's correlation test (Pearson correlation = −0.169, *P* = 0.01). We also found tumors with lower T stage, negative surgical margin, and negative capsule invasion expressed a higher level of cytoplasmic CCND1 as well, which indicated that cytoplasmic CCND1 could be a biomarker of lower grade and less invasive potential PCa. On the basis of the Ki-67 analysis in Comstock's study, we further proved the protective prognostic value of cytoplasmic CCND1 (*P* = 0.002) in the BCR-free time analysis. However, after adding other clinicopathologic features, the cytoplasmic CCND1 expression level was excluded in the multivariate Cox proportional hazard regression analysis (*P* = 0.242Table 3), which means the prognostic value cytoplasmic CCND1 is still limited.

Unlike Comstock's work, another study based on 50 samples [10], cytoplasmic CCND1 showed an expression increasing with the Gleason grade (3, 4, and 5). And another study focused on lymph node metastases PCa demonstrated that the cytoplasmic CCND1 in primary tumor had no correlation with any tumor feature or survival [19]. The differences among existing researches could result from those studies focused on different stages of the tumor. The most obvious difference between our study and other studies is that we found cytoplasmic CCND1 was higher in adjacent normal tissue, while other studies showed the opposite, which could result from the overall disease stage of patients and mostly, the standard of evaluating cytoplasmic CCND1 (our score was based on the IE score while other studies were based on the positive proportion of cytoplasmic CCND1). Other reasons including sample size, race difference, and precision of evaluating strategies for IHC could also lead to the difference among current reaches. For further validating our results, we analyzed two GEO databases (GSE21034, GSE62872) indicating that the CCND1 mRNA level is higher in non-PCa samples (*P* < 0.001). A previous PCa sequencing data of our institution showed CCND1 mRNA level was higher in adjacent normal tissue than tumor tissue (*P* < 0.001) which further confirmed the cytoplasmic CCND1 protein expression level results of IHC data in this study. However, no correlation was found between CCND1 mRNA level and any tumor feature in those databases.

It has been reported that CCND1 is synthesized in the cytoplasm and assembled with CDK4 and CDK6 after been stimulated by several mitogenic signals. After been activated by phosphorylation, the CCND1-CDK complex enters the nucleus by active transport or via nuclear pores [20, 21], and this complex regulates the cell cycle by phosphorylating (1) S-phase gene expression negative regulators and (2) a class of Cdk inhibitors [8, 22]. In our study, although the CCND1 mRNA level and cytoplasmic CCND1 were both found higher in adjacent normal tissue, nuclear CCND1 was found higher in tumor tissue. And this result could be a circumstantial evidence of the nucleocytoplasmic transport of CCND1, suggesting that despite the total expression of CCND1 was more abundant in normal tissues and concentrated in cytoplasm, CCND1 in tumor cells was more likely to be transported and accumulated in the nucleus. However, the reason of CCND1 was enriched in the cytoplasm of normal tissues still needs further study.

Unlike cytoplasmic CCND1, nuclear CCND1 has been well studied in several articles. Michail et al. [15] claimed that along with other three biomarkers (PTEN, SMAD4, and SPP1), nuclear CCND1 (HR = 1.99, *P* = 0.036) can be a valuable biomarker to predict lethal outcome of PCa. Similarly, Ding et al. [16] found that along with PTEN, SMAD4, and SPP1, CCND1 could enhance the prognostic value of Gleason score in predicting BCR and lethal outcome. However, in a follow-up study with a median follow-up time of more than 10 years, Ding et al. [17] overturned their own conclusion that those markers cannot improve the prognosis value of clinical factors. In another study, Anthony et al. [18] found that nuclear CCND1 alone was significantly associated with BCR (*P* = 0.042, HR = 1.38), while after adjusting clinicopathologic features in the COX proportional hazards regression model, the significance of nuclear CCND1 descended (*P* = 0.296) and this index was excluded. In our study, the nuclear CCND1 expression level did not show any correlation with the time to BCR (*P* = 0.286), and the combination of cytoplasmic and nuclear CCND1 did not show a better prognostic value than the cytoplasmic CCND1 alone either. Thus, combining with previous studies, the prognostic value of nuclear CCND1 is very limited in PCa.

There are several limitations should be considered in this study. First, the study was a single-center study and the sample size is limited, although several online databases were considered in this study, a study basing on different medical centers can be more convincing. Second, this study found that CCND1 was enriched in the cytoplasm of normal tissues in PCa by TMA and online data base analysis; however, the reason of this phenomenon and the biological function of cytoplasmic CCND1 still needs further study.

## 5. Conclusion

This study showed that adjacent normal tissues had higher CCND1 expression in than prostate cancer tissues in mRNA and cytoplasmic protein level, and perhaps due to nucleo-cytoplasmic transport, nuclear CCND1 showed opposite distribution characteristic. We also found that cytoplasmic CCND1 rather than nuclear CCND1 has a protective prognostic value in the BCR-free time analysis and has correlation with several clinical indicators.

## Figures and Tables

**Table 1:** The clinical and pathological characteristics of patients.

Variables	*n*	Percentage(%)
Age (year)		
<60	28	14.89%
60-70	83	44.15%
>70	77	40.96%
pT stage		
pT2	113	60.11%
pT3+pT4	75	39.89%
Gleason score		
≤6	20	10.64%
7	89	47.34%
≥8	79	42.02%
Preoperative PSA level (ng ml-1)		
<4	3	1.60%
4–10	36	19.15%
10–20	70	37.23%
>20	79	42.02%
pN stage		
p N0	156	82.98%
p N1	32	17.02%
Surgical margin		
Positive	82	43.62%
Negative	106	56.38%
Prostate capsule invasion		
Positive	68	36.17%
Negative	120	63.83%
Seminal vesicle invasion		
Positive	42	22.34%
Negative	146	77.66%
Nerve invasion		
Positive	81	43.09%
Negative	107	56.91%

**Table 2:** Cytoplastic and nuclear CCND1 expression status in TMA.

Variables	Samples	Percentage	Cytoplasmic CCND1		*P*	Nuclear CCND1		*P*
			Weak	Strong		Weak	Strong	
Tissue type					<0.001			<0.001
Prostate cancer	188	50.00%	103	85		82	106	
Adjacent tissue	188	50.00%	53	135		117	71	
pT stage					<0.001			0.931
pT2	113	60.11%	50	63		49	64	
pT3+pT4	75	39.89%	53	22		33	42	
Gleason score					0.028			0.4
≤6	20	10.64%	8	12		11	9	
7	89	47.34%	43	46		35	54	
≥8	79	42.02%	52	27		36	43	
Preoperative PSA level (ng ml-1)					0.142			0.63
<4	3	1.60%	1	2		1	2	
4–10	36	19.15%	30	6		19	17	
10–20	70	37.23%	37	33		28	42	
>20	79	42.02%	50	29		34	45	
pN stage					0.177			0.425
p N0	156	82.98%	82	74		66	90	
p N1	32	17.02%	21	11		16	16	
Surgical margin					0.037			0.945
Positive	82	43.62%	52	30		36	46	
Negative	106	56.38%	51	55		46	60	
Prostate capsule invasion					0.04			0.682
Positive	68	36.17%	44	24		34	34	
Negative	120	63.83%	59	61		51	69	
Seminal vesicle invasion					0.294			0.811
Positive	42	22.34%	26	16		19	23	
Negative	146	77.66%	77	69		63	83	
Nerve invasion					0.285			0.324
Positive	81	43.09%	48	33		32	49	
Negative	107	56.91%	55	52		50	57	

**Table 3:** Univariate and multivariate analyses of CCND1 expression in BCR-free survival time analysis.

Univariate	BCR-free survival time	
	HR (95% CI)	*P*
Low IE score (cytoplasmic)	1	
High IE score (cytoplasmic)	0.536 (0.362-0.793)	0.002
Low IE score (nuclear)	1	
High IE score (nuclear)	0. 816 (0.561-1.186)	0.286
Multivariate		
IE score (cytoplasmic) (low/high)	0.774 (0.504-1.189)	0.242
pT stage (pT2/pT3, pT4)	1.736 (1.128-2.672)	0.012
pN stage (pNx, pN0/pN1)	2.560 (1.576-4.159)	<0.001
Gleason score (<4 + 4/≥4 + 4)	2.011 (1.337-3.026)	0.001
PSA level (>10/≤10)	2.096 (1.147-3.829)	0.016

## Data Availability

The TMA immunohistochemical staining data used to support the findings of this study are included within the article, and the online database could be accessed at https://www.ncbi.nlm.nih.gov/geo/.

## Supplementary Materials

Supplementary Materials Figure S1: relationship between different cytoplastic and nuclear CCND1 expression patterns and BCR-free survival time. Read less.
