# Supplementary figures and images for: Differential Expression and Prognostic Value of Cytoplasmic and Nuclear Cyclin D1 in Prostate Cancer
Source: Biomed Res Int. 2020 May 30;2020:1692658. doi: 10.1155/2020/1692658 (PMC7281841; doi:10.1155/2020/1692658)

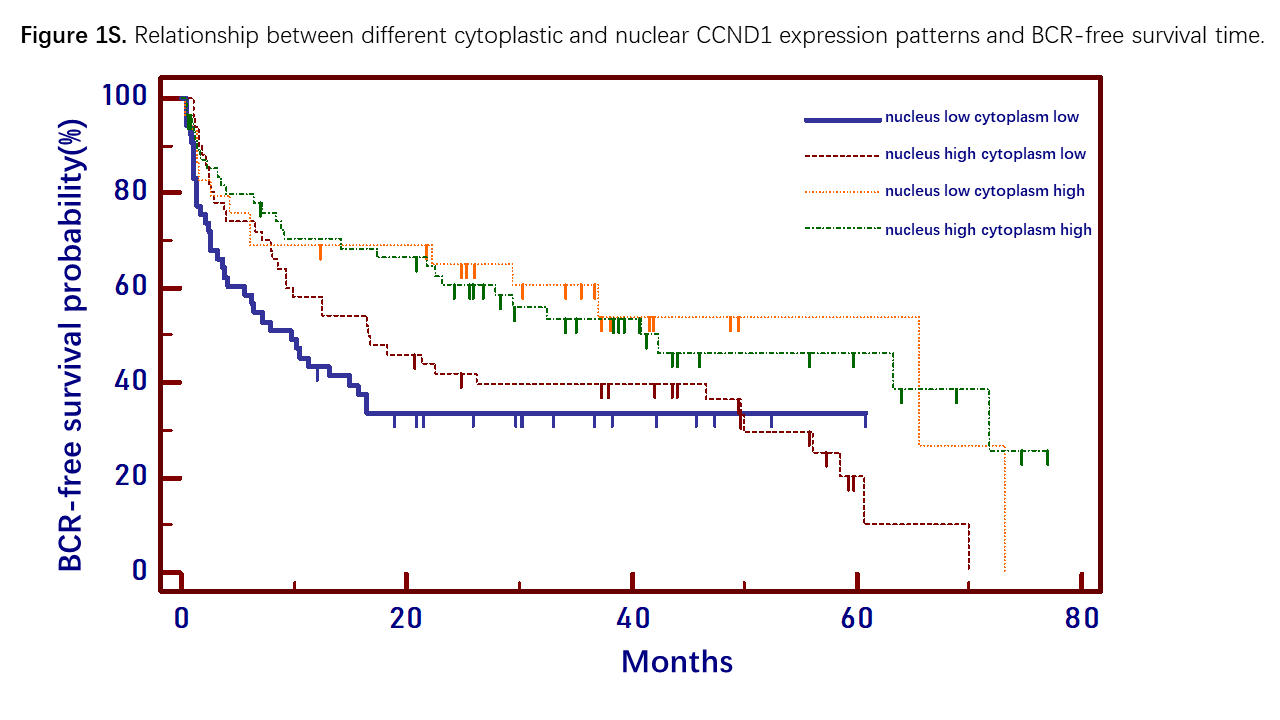

Supplement: Supplementary Materials — Figure S1: relationship between different cytoplastic and nuclear CCND1 expression patterns and BCR-free survival time. Read less. [file 1692658.f1.tif]
